# Density Functional Theory Approach to the Vibrational Properties and Magnetic Specific Heat of the Covalent Chain Antiferromagnet KFeS_2_

**DOI:** 10.3390/molecules27092663

**Published:** 2022-04-20

**Authors:** Airat Kiiamov, Maxim Kuznetsov, Dorina Croitori, Irina Filippova, Vladimir Tsurkan, Hans-Albrecht Krug von Nidda, Zakir Seidov, Franz Mayr, Sebastian Widmann, Farit Vagizov, Dmitrii Tayurskii, Lenar Tagirov

**Affiliations:** 1 Institute of Physics, Kazan Federal University, 420008 Kazan, Russia; mdkuznecov@stud.kpfu.ru (M.K.); vagizovf@gmail.com (F.V.); dmitry.tayurskii@kpfu.ru (D.T.); ltagirov@mail.ru (L.T.); 2 Institute of Applied Physics, MD-20208 Chisinau, Moldova; dorinacroitori@gmail.com (D.C.); irina.filippova@phys.asm.md (I.F.); vladimir.tsurkan@physik.uni-augsburg.de (V.T.); 3 Experimental Physics V, Center for Electronic Correlations and Magnetism, Institute of Physics, University of Augsburg, D-86135 Augsburg, Germany; hans-albrecht.krug@physik.uni-augsburg.de (H.-A.K.v.N.); zsyu@rambler.ru (Z.S.); franz.mayr@physik.uni-augsburg.de (F.M.); sebastian.widmann@physik.uni-augsburg.de (S.W.); 4 Institute of Physics, Azerbaijan National Academy of Sciences, AZ-1143 Baku, Azerbaijan; 5 Zavoisky Physical-Technical Institute, FRC Kazan Scientific Center of RAS, 420029 Kazan, Russia

**Keywords:** ternary potassium-iron sulfide, quasi-one-dimensional antiferromagnet, density functional theory, phonon density of states, magnetic specific heat, spin chain models

## Abstract

Ternary potassium-iron sulfide, KFeS_2_, belongs to the family of highly anisotropic quasi-one-dimensional antiferromagnets with unusual “anti-Curie–Weiss” susceptibility, quasi-linearly growing with a rising temperature up to 700 K, an almost vanishing magnetic contribution to the specific heat, drastically reduced magnetic moment, etc. While some of the measurements can be satisfactorily described, the deficiency of the entropy changes upon the magnetic transition and the spin state of the iron ion remains a challenge for the further understanding of magnetism. In this work, high-quality single-crystalline samples of KFeS_2_ were grown by the Bridgman method, and their stoichiometry, crystal structure, and absence of alien magnetic phases were checked, utilizing wave-length dispersive X-ray electron-probe microanalysis, powder X-ray diffraction, and ^57^Fe Mössbauer spectroscopy, respectively. An ab initio approach was developed to calculate the thermodynamic properties of KFeS_2_. The element-specific phonon modes and their density of states (PDOS) were calculated applying the density functional theory in the DFT + U version, which explicitly takes into account the on-site Coulomb repulsion U of electrons and their exchange interaction J. The necessary calibration of the frequency scale was carried out by comparison with the experimental iron PDOS derived from the inelastic nuclear scattering experiment. The infrared absorption measurements confirmed the presence of two high-frequency peaks consistent with the calculated PDOS. The calibrated PDOS allowed the calculation of the lattice contribution to the specific heat of KFeS_2_ by direct summation over the phonon modes without approximations and adjustable parameters. The temperature-dependent magnetic specific heat evaluated by subtraction of the calculated phonon contribution from the experimental specific heat provides a lower boundary for estimating the reduced spin state of the iron ion.

## 1. Introduction

**Figure 1:**
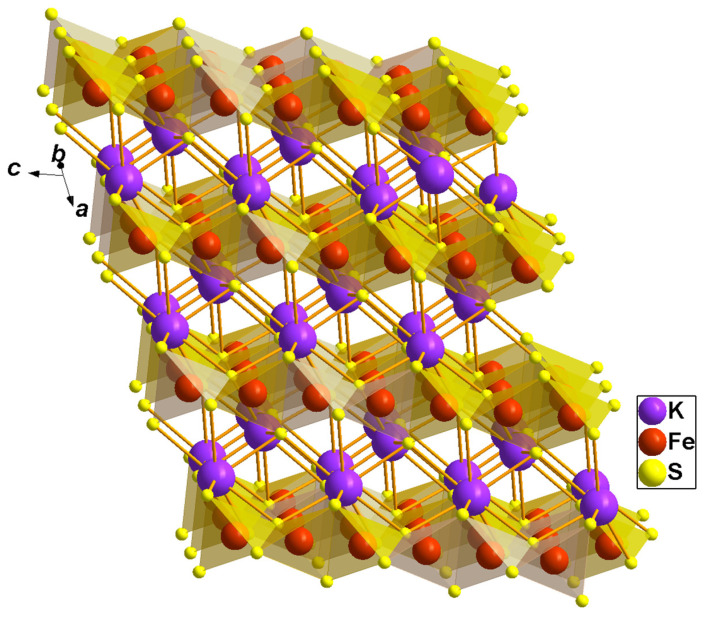
Crystal structure of KFeS_2_. The FeS_4_ tetrahedra, with Fe drawn as a red sphere in the center and S as a yellow sphere at the corners and highlighted in a transparent orange color. Violet large spheres denote K.

Ternary potassium-iron sulfide, KFeS_2_, belongs to the family of quasi-one-dimensional compounds with the general chemical composition *A*Fe*Ch*_2_ (where *A*–K or Rb; *Ch*–S or Se) [1,2]. These compounds share a common structural motif, i.e., a chain of edge-sharing chalcogen tetrahedra with iron ions inside (see Figure 1) [1,2,3]. Because of the strong intra-chain antiferromagnetic exchange interaction between the iron ions, these compounds are considered as model ones to study the magnetic properties and thermal behavior of a one-dimensional antiferromagnetic chain. KFeS_2_ is the most extensively investigated compound of this family. Nevertheless, so far, no consensus has been reached on the magnetic ground state of this compound, and the magnetic moment values presented in the literature are different from each other [4,5,6,7]. Several investigations argue that the spin state of the iron ions in this compound is low, *S* = 1/2 [4,5]. Therefore, in [6], it was noticed that the spin-1/2 one-dimensional Heisenberg model provides a good fit for the temperature dependence of the susceptibility of KFeS_2_. In contrast, based on the X-ray absorption spectroscopy study, the spin state was estimated as *S* = 5/2 [7]. Moreover, the magnetic moment of the iron ions in KFeS_2_ of about 1.9 μ_B_ obtained from neutron diffraction studies in [1] suggests that the intermediate spin state *S* = 3/2 is quite probable.

Neutron diffraction and heat capacity studies [1,8,9] find the compound as an antiferromagnet with a Néel temperature of 250 K. The spins of the iron ions are antiferromagnetically coupled along the chain, thereby creating alternating ferromagnetic sheets [1]. However, the magnetic order of the compound is not fully established. Another study [10], based on magnetic susceptibility measurements, a molecular-field approximation, and the temperature-dependent Green’s function method, suggests that in KFeS_2_ the three-dimensional antiferromagnetic state sets in at a Néel temperature of 12.5 K rather than at 250 K, as is usually assumed. Hence, it was proposed to perform low-temperature total and magnetic specific heat measurements to further elucidate the nature of the magnetic ordering and the values of the exchange constants in this interesting quasi-one-dimensional system [10].

The temperature dependence of the heat capacity of the magnetic subsystem of KFeS_2_ could also shed light on the problem of estimating the Néel temperature and spin state of the iron ions in this compound. If the magnetic contribution to the heat capacity is determined over a wide temperature range, the change in the entropy of the magnetic subsystem contained in the transition from complete magnetic disorder in the paramagnetic state at high temperatures to an ordered state at low temperatures can be estimated and then compared with the values that are predicted for specific spin states.

The standard approach associates the magnetic contribution to the total heat capacity with the lambda anomaly at the temperature of the onset of magnetic order. The temperature dependence of the heat capacity beyond the lambda anomaly is usually treated as the lattice contribution, which can be fitted by a combination of Debye and Einstein contributions. The resulting difference between the total measured specific heat and the modeled lattice specific heat is ascribed to the magnetic contribution. However, sometimes in the case of a weak lambda anomaly, the accumulated entropy change appears to be an order of magnitude smaller than the lowest one, *Rln*2 = 5.76 J mol^−1^ K^−1^, expected when assuming an *S* = 1/2 state of Fe^3+^ [9,11,12,13,14,15,16,17].

In [17], doubts were expressed about the applicability of the standard description of phonons in compounds with a complex unit cell and significant anisotropy in the framework of the Debye and Einstein models. Indeed, ab initio calculations for phonon spectra and their density of states (PDOS) in the quasi-one-dimensional chain compound RbFeSe_2_ have shown a complicated structure of PDOS [16]. After summation over all phonon modes in the entire frequency range, the calculated lattice heat capacity appeared to be significantly different from the phenomenological one. It was shown that the magnetic contribution to the heat capacity, taken as a difference between the measured and calculated ones, extends from zero temperature to temperatures far above the Néel temperature of 250 K. The associated change in the magnetic entropy required a reasonable low-boundary value Δ*S_M_* = 6.03 J mol^−1^ K^−1^, which is in between the 5.76 J mol^−1^ K^−1^ and 11.52 J mol^−1^ K^−1^ expected for the *S* = 1/2 and *S* = 3/2 spin states, respectively. However, the temperature limit of 290 K of our equipment did not allow us to give an unambiguous answer about the spin state of the Fe^3+^ ions in RbFeSe_2_.

Applying the above-mentioned approach to KFeS_2_, it can be assumed that an accurate analysis of the temperature dependence of the heat capacity can make progress in resolving the existing contradictions in studies regarding the magnetic properties of this compound. To start with, we notice that KFeS_2_ is a poor conductor with resistivity above 10^3^ Ω∙cm at temperatures below 250 K [18] and a semiconductor-type temperature dependence towards low temperatures. This allows the contribution of the charge carriers to the specific heat to be neglected.

Ab initio density functional theory calculations allow calculation of the phonon frequencies and their density of states [19,20]. Nevertheless, due to systematic over- or underestimation of inter-ion forces, it is known that the frequency scale of the calculated phonon density of states (PDOS) needs to be corrected [20,21].

Some experimental methods, sensitive and susceptible to the vibrational properties of solids, such as infrared (IR) absorption, Raman spectroscopy, Mössbauer spectroscopy, nuclear inelastic scattering, neutron inelastic scattering, etc., can be used for calibration of the calculated PDOS. Our calibration for RbFeSe_2_ was realized using IR absorption and Mössbauer spectroscopy techniques [18]. It was an implicit procedure from a qualitative comparison with the frequency dependence of the IR absorption intensity and the best fit of the temperature dependence of the Lamb–Mössbauer factor. In the present work, the nuclear inelastic scattering (NIS) method yields the partial phonon density of states in an output that can be directly compared with the calculated one. This allows the necessary frequency correction factor to be found [22,23,24]. The only limitation of this method is that the compound must contain Mössbauer nuclei, which is the case in our study (Fe in KFeS_2_).

The goals of the present study were: (i) to synthesize high-quality single-crystalline samples of KFeS_2_, (ii) to perform ^57^Fe nuclear inelastic scattering measurements to obtain the partial PDOS of the iron ions in this compound, (iii) to perform IR-absorption measurements, (iv) to measure the total specific heat of the single-crystalline samples of KFeS_2_, (v) to calculate the total and partial phonon densities of states within the DFT + U approach and check their agreement with NIS and IR-absorption experimental data, (vi), to calculate from total PDOS the lattice contribution to the specific heat of KFeS_2_, and (vii), eventually, to obtain the temperature dependence of the magnetic specific heat of KFeS_2_ as the difference between the total heat capacity and the lattice specific heat.

## 2. Synthesis, Structural Characterization, and Experimental Methods

**Figure 2:**
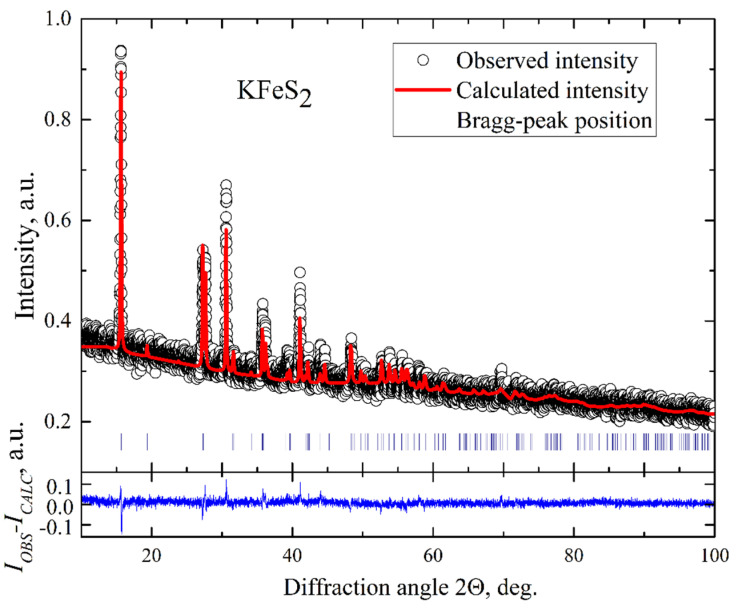
Powder X-ray diffraction pattern of KFeS_2_. The empty circles represent the measured intensities. The solid red line is the refined pattern. The Bragg peak positions are indicated by vertical blue bars. The blue line represents the difference between the measured spectrum and the fit.

KFeS_2_ single crystals were grown using the Bridgman method. They had a needle-like shape as was observed for the related compound RbFeSe_2_ [18]. Such a shape indicates the quasi-one-dimensional structure of these compounds. The elemental composition was determined using wave-length dispersive X-ray electron-probe microanalysis (WDX EPMA, Cameca SX50, Industriële Veiling Eindhoven, Eindhoven, the Netherlands), which was 24.72 at% for K, 24.72 at% for Fe, and 50.56 at% for S, respectively, resulting in the composition of K_0.99_Fe_0.99_S_2.02_, indicating insignificant deviation from the exact stoichiometry of KFeS_2_. The structural parameters of the crystals were investigated employing conventional X-ray diffraction (XRD) on powdered single crystals at room temperature using a STOE STADI P diffractometer (Darmstadt, Germany) with Cu-K_α_ radiation. A typical XRD pattern of our KFeS_2_ sample is displayed in Figure 2. The pattern reveals a narrow width of peaks that indicates a good quality of the crystals. The data were analyzed by a standard Rietveld refinement using the FullProf software [25]. The XRD pattern was described within a quasi-one-dimensional monoclinic structure with C2/c space symmetry group and lattice parameters *a* = 7.092(2) Å, *b* = 11.348(2) Å, *c* = 5.398(2) Å, and *β* = 113.2(1)°. This description correlates well with the crystallographic data reported for KFeS_2_ using neutron diffraction at room temperature [1]. Since all diffraction peaks were well described within the known KFeS_2_ structure model, it can be argued that the samples contain no impurity phases. The refinement does not fully describe the observed peak intensities. It can result from a combined effect of strain of the sample when crushed into powder and texture. Thus, from the microprobe and XRD studies, we conclude that the composition of our KFeS_2_ samples exhibits only small deviations from the stoichiometry and no foreign phases.

The data analysis reveals two short (2.097 Å) and two long (2.299 Å) Fe-S distances, as well as four different S-Fe-S angles, which indicate a strong distortion of the FeS_4_ tetrahedra. The intra-chain Fe-Fe distance of 2.699 Å exceeds the Fe-Fe distance in metallic iron by about 9%.

To exclude the possibility of sample contamination caused by iron-containing impurities with an amount below what can be detected by microprobe and XRD, we used conventional Mössbauer spectroscopy to characterize the samples. Because of strong magnetism of iron, these contaminants can complicate and compromise an accurate analysis of the magnetic properties of theKFeS_2_samples. The Mössbauer spectra were recorded at room temperature and a temperature of 4.2 K on a conventional constant acceleration spectrometer (Wissel, Germany) equipped with a room-temperature rhodium-matrix cobalt-57 gamma-radiation source. The observed spectra were least-square fitted with the assumption that the line shapes are Lorentzian. Beforehand, the spectrometer was calibrated at room temperature with an α-iron foil.

**Figure 3:**
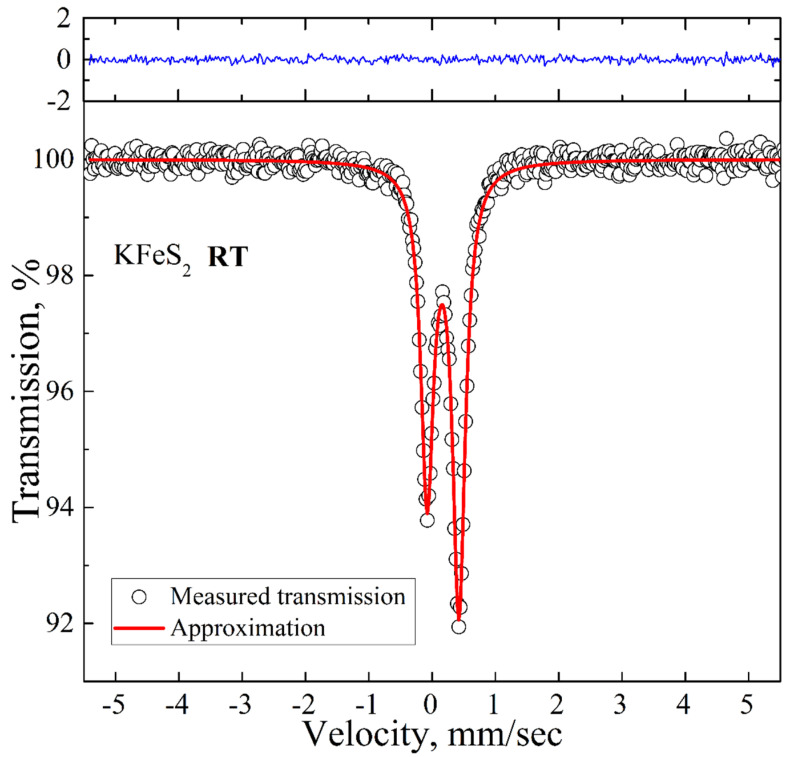
Room temperature ^57^Fe Mössbauer spectrum of KFeS_2_ (empty circles). Solid red lines represent the best fitting of the spectrum obtained by the least-squares fit (red line) under the assumption that the line shapes are Lorentzian. The blue line represents the difference between the measured spectrum and the fit.

The room temperature spectrum is presented in Figure 3. It is an asymmetric doublet with the QS and IS values of 0.50 mm/s and 0.17 mm/s, respectively, which are in good agreement with the previously reported Mössbauer data for KFeS_2_ [26]. The asymmetry of the spectrum is a consequence of the quasi-one-dimensional structure of KFeS_2_ and the needle-like shape of the samples, which complicates the preparation of a fully isotropic powder sample. We did not detect any additional peaks above the background.

**Figure 4:**
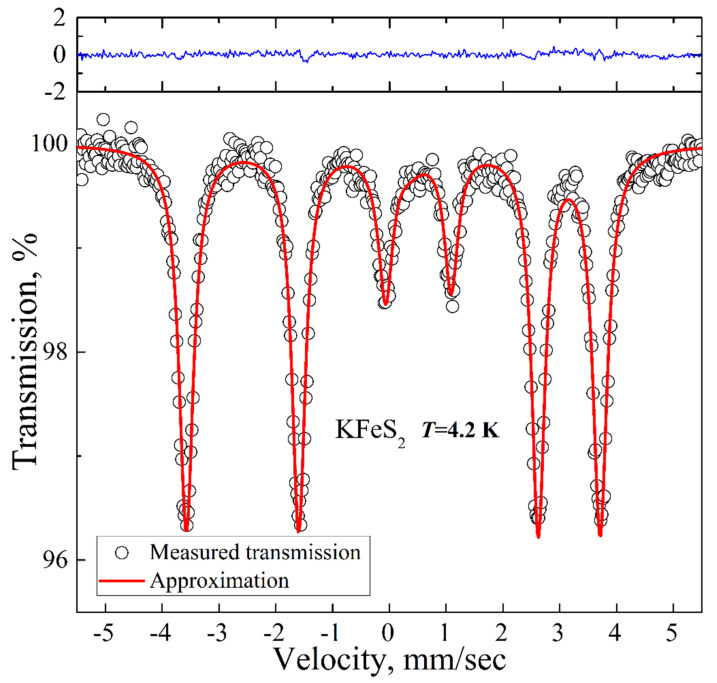
Low-temperature ^57^Fe Mössbauer spectrum of KFeS_2_ (empty circles). Solid red lines represent the best fitting of the spectrum obtained by the least-squares fit (red line) under the assumption that the line shapes are Lorentzian. The blue line represents the difference between the measured spectrum and the fit.

The low-temperature spectrum is presented in Figure 4. It is a magnetic sextet with the QS, IS, and HF values of 0.57 mm/s, 0.09 mm/s, and 225 kOe, respectively. The experimental spectrum is well described within a model with a single contribution of a magnetic sextet due to the long-range magnetic order already established in the compound at this temperature. The hyperfine field value of 225 kOe is in good agreement with the reported previous results on KFeS_2_ [27] (i.e., 237 kOe extrapolated to *T* = 0 K).

The hyperfine parameters of KFeS_2_ correlate well with the Mössbauer results for the related iron chalcogenide RbFeSe_2_ [17]. The value of the hyperfine field is significantly smaller than the values for the high and intermediate spin states of Fe^3+^ in ionic oxide compounds (about 500 kOe and 300 kOe, respectively [28]). Such a low value of the hyperfine field indicates a strong reduction in the local spin moment of the iron ions. We should note that both room temperature and low-temperature spectra are well described by models with a single contribution of an asymmetric doublet and a magnetic sextet, respectively. Both model spectra are in good quantitative agreement with the previously reported results on KFeS_2_. Summarizing the XRD and Mössbauer spectroscopy results, we conclude that our sample is a high-quality KFeS_2_ crystal without iron-containing impurity phases.

The NIS [22,23] experiment was carried out by the Dynamics Beamline P01 of PETRA III synchrotron (DESY, Hamburg, Germany) [29]. The measurements utilizing the nuclear gamma-resonance of ^57^Fe at 14.413 keV were performed with an inline high-resolution monochromator providing an energy bandwidth of 0.9 meV full width at half maximum (FWHM). The sample with a natural abundance of ^57^Fe was measured at 295 K.

The IR absorption was obtained in the frequency range 4–14 THz at a Bruker IFS 113v spectrometer (Bruker Analytik GmbH, Karlsruhe, Germany). KFeS_2_ was ground and dispersed in CsI powder in the ratio of 1.35 mg:100 mg. This mixture was cold-pressed under vacuum to obtain a disk-like pellet with a diameter of 13 mm.

The specific heat of single-crystalline samples of KFeS_2_ was measured by a relaxation method using a PPMS (Quantum Design) in the temperature range 1.8 ≤ *T* ≤ 300 K.

## 3. Experimental Results

**Figure 5:**
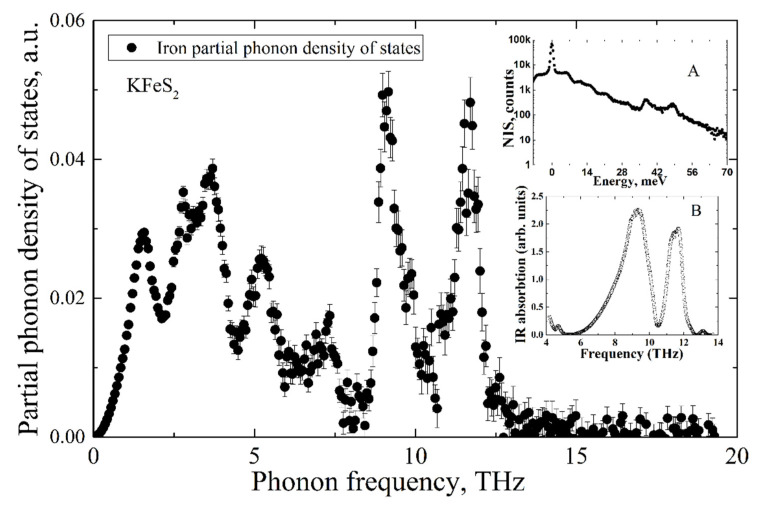
Partial iron PDOS of KFeS_2_ evaluated from the nuclear inelastic scattering spectrum of KFeS_2_ (presented in the inset A) and IR-absorption spectrum (presented in inset B).

The Fe NIS spectrum of KFeS_2_ is shown in inset A of Figure 5. The partial Fe PDOS was evaluated from the NIS spectrum using the procedure described in [23] and is presented in the mainframe of Figure 5. The experimental infrared absorption spectrum is depicted in inset B of Figure 5.

While the IR-absorption spectrum allows estimation of the non-zero PDOS only for optical phonon modes, the NIA technique measures the density of states of all phonon modes but only for iron ions. As is predicted by the classical Debye model, in the low-frequency range, the partial PDOS of iron ions depends on vibrational frequency by cubic law.

Notably, both methods of NIS and IR-absorption show two peaks of high intensity in the density of states of optical phonons in the frequency ranges of 8–10 THz and 10–12 THz. Such a good agreement of two independent methods allows for arguing for the high accuracy of the results.

## 4. Density Functional Theory Calculations for the Phonon Density of States of KFeS_2_

Density functional theory (DFT) ab initio calculations were performed by means of the Vienna Ab initio Simulation Package (VASP 5.4) [30,31,32,33]. The electron–ion interactions were taken into account using the projector-augmented wave (PAW) method, i.e., a frozen-core method including the exact shape of the wave functions of the valence electrons instead of pseudo-wave functions [34]. The Perdew–Burke–Ernzerhof (PBE) generalized gradient approximation (GGA) was applied for the exchange and correlation corrections [35]. The K (3p^6^ 4s^1^), Fe (3d^6^ 4s^2^), and S (3s^2^ 3p^4^) electrons of the valence shell were treated explicitly, whereas the remaining electrons of the cores were taken into account by using pseudopotentials. The maximum energy for the plane-wave basis was selected to be equal to 500 eV. The k-point mesh was a 5 × 5 × 6 Monkhorst–Pack grid, which corresponds to the actual spacing of 0.227 × 0.228 × 0.221 per Å [36]. Equilibrium geometry was obtained after several stages of full structural relaxation including atomic positions, cell shape, and cell volume. The force tolerance was 0.2 eV/nm and the energy tolerance for the self-consistency loop was 10^−5^ eV. The phonon dispersion and density of states were obtained utilizing a direct approach of harmonic approximation making use of the MedeA Phonon commercial software [37,38]. The so-called direct approach to the lattice dynamics is based on the ab initio evaluation of forces on all atoms by a set of finite displacements of a few atoms within an otherwise perfect crystal. The phonon frequencies are calculated from the eigenvalues of the resulting dynamical matrix. The totally optimized equilibrium crystal structure was used for the calculation of the phonon dispersion. The lattice parameters obtained after the lattice relaxation are given by *a* = 6.89 Å, *b* = 11.02 Å, *c* = 5.10 Å, and the angle *β* = 111.56°. A slight deviation in the calculated lattice parameters from the experimental ones [1], of about 1–5 percent, is typical for DFT calculations [39].

All calculations accounted for the spin polarization due to the antiferromagnetic state of KFeS_2_. The antiferromagnetic spin pattern was set in accordance with the magnetic structure obtained previously by neutron diffraction data [13].

We published the calculated PDOS of KFeS_2_ within the DFT approach in [40]. The partial PDOS of the iron ions is not in good agreement with the NIS experimental data presented in the current study (Figure 5). The PDOS published in [40] was obtained with preliminary calculations within a simple DFT approach, which ignores any Hubbard-like U parameter [41]. It was mentioned that the magnetic moment of iron ions obtained in [40] is not in agreement with the neutron diffraction experimental results [1]. This may point to the strong delocalization of Fe *d*-electrons, and the DFT + U approach is necessary for a proper description of the system [40,42].

A simplified DFT approach corrects some of the inadequacies connected to the DFT treatment of localized states but suffers from the dependence of the results on the value of U_eff_ = U − J (the difference between intra-atomic Coulomb and exchange energies [43]), which de facto is an empirical parameter [44].

In [42], it was shown that the set of parameters U = 1.5 eV and J = 2 eV (U_eff_ = −0.5 eV) provides a correct description of the magnetic properties (magnetic moment) and electronic properties (bandgap value) of KFeS_2_ simultaneously. Thus, the calculations presented in this paper were carried out within the DFT + U scheme with the above-mentioned set of parameters.

**Figure 6:**
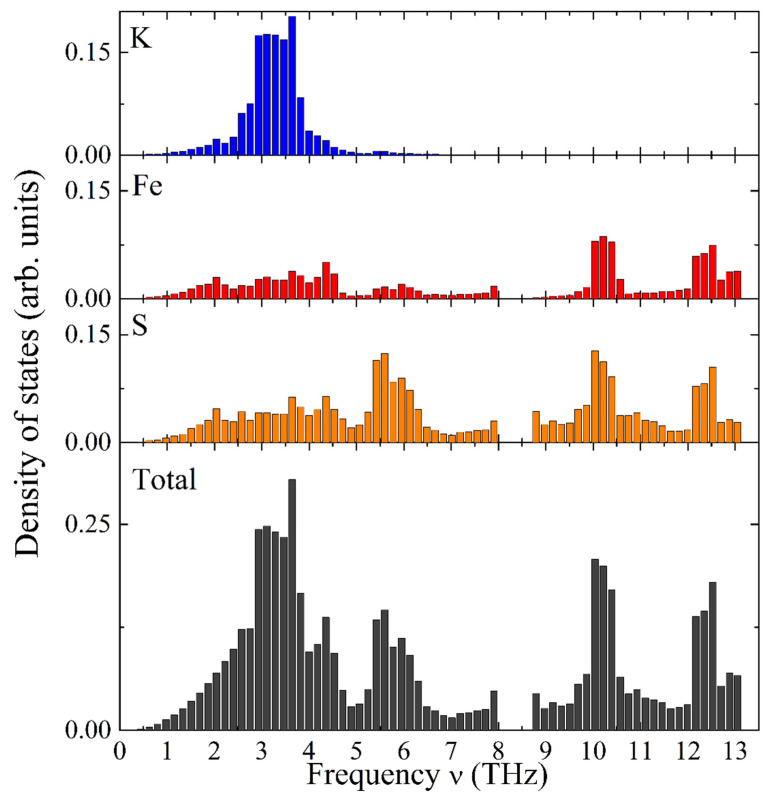
Calculated PDOS as a function of frequency in KFeS_2_: element-specific (K, Fe, and S atoms from top towards bottom) and the total PDOS (bottom).

Figure 6 illustrates the calculated total and element-specific PDOS as a function of frequency for KFeS_2_. It exhibits a complex structure distributed over two distinct frequency ranges. The vibrational modes of potassium atoms have relatively low frequencies, thus dominating the frequency range of 1–5 THz (see Figure 6, the top bar chart in blue color). Iron atoms possess the highest vibrational frequencies and constitute a substantial part of the high-frequency range (9–13 THz, see Figure 6, the second bar chart from the top in red color). Sulfur atoms show vibrational modes in the whole frequency range under consideration (see the third bar chart from the top of Figure 6). The Debye-type PDOS of the K atoms manifests vibrations almost independently of the iron atoms in the chain, as expected for weakly bound K atoms.

One more feature of the calculation method needs to be clarified before we pass to the temperature dependence of the specific heat. As we mentioned and have shown in [18], the DFT approach usually under- or overestimates vibrational frequencies of the phonon modes. A frequency correction factor is usually established by comparison of the calculated and experimental phonon spectra [18]. In the present study, the factor is obtained from a comparison of the calculated results with the experimental data on NIS and IR absorption.

**Figure 7:**
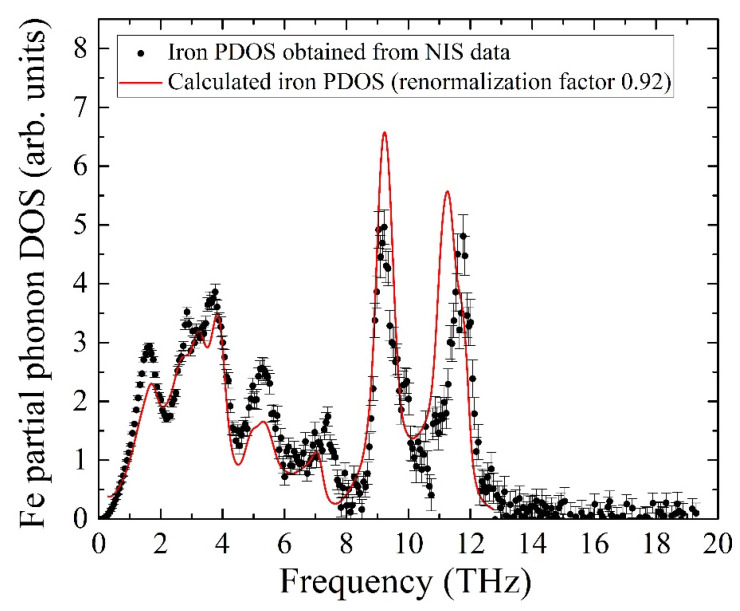
Partial PDOS for iron atoms of KFeS_2_ obtained from the NIS spectrum (black dots) and from ab initio calculations (red line, see description in the body text).

The calculated PDOS is a discrete set of contributions from phonon modes, while the experimental data is a quasi-continuous sequence determined by the settings of the beamline setup, i.e., mainly by the monochromator resolution of 0.9 meV. To compare the experiment with the ab initio calculated iron partial PDOS, the latter was convoluted with a Gaussian profile with an FWHM of 0.9 meV, matching the resolution window of the monochromator. The comparison of the convolution with the experimental data is presented in Figure 7 and shows adequate agreement between the PDOS evaluated from our NIS data and the ab initio calculated PDOS with the frequency scale corrected by a factor of 0.92. The calculated pattern quantitatively describes all features of the iron PDOS within the entire frequency range of vibrations in the KFeS_2_ lattice.

The insignificant disagreement in amplitudes should be the result of a fast oxidation tendency of KFeS_2_ in the air atmosphere, which must lead to crystal structure defects. The considered sample was exposed to air for about 10 min on the probe preparation, and during the measurements, the sample was protected by Kapton scotch tape, which does not provide the best protection. Moreover, the theoretical PDOS is calculated for the case of a fully isotropic sample, while KFeS_2_ consists of small needle-like crystallites in the powdered sample that prevents the preparation of an isotropic sample. The last fact is important because the PDOS of KFeS_2_ is anisotropic.

**Figure 8:**
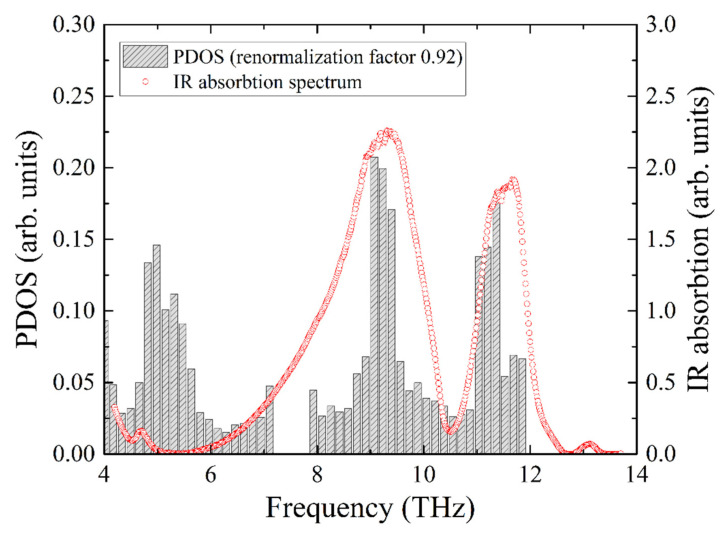
Infrared absorption spectrum of KFeS_2_ compared with the calculated PDOS of KFeS_2_. The frequency scale of the PDOS is corrected to obtain the best coincidence of the peaks of PDOS maxima with the IR absorption maxima (shaded bar charts).

The experimental infrared absorption spectrum shows two broad lines, which are compared with the total PDOS in Figure 8. The best agreement for the resonant frequencies of the two experimental absorption peaks with the calculated ones was obtained for the frequency correction factor of 0.92. Notably, this is the same value that we obtained from the comparison of the iron PDOS with the NIS data.

## 5. Specific Heat Analysis

**Figure 9:**
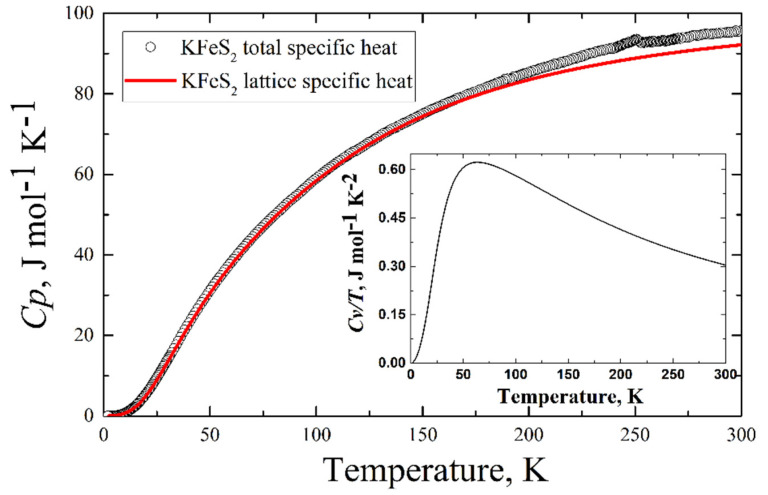
Temperature dependence of the specific heat *C_P_* of KFeS_2_ (black circles)—experimental data, red line—calculated lattice contribution to the specific heat at constant pressure *C_P_* = *C_V_* + 0.003 [J/mol K^2^]*T*. Inset—the calculated specific heat at constant volume drawn as *C_V_*(*T*)/*T*.

The experimentally measured total specific heat C(T) of the KFeS2 single crystal is presented in Figure 9 (see the legend). An anomaly is observed in the C(T) data at TN = 247 K. The phonon contribution to the heat capacity reaches a value of 95 J mol^−1^ K^−1^ at room temperature. This value does not exceed Dulong–Petit law’s limit (about 99 J mol^−1^ K^−1^).

The total specific heat is assumed to originate from two contributions: a lattice contribution due to acoustic and optical phonons, and a magnetic contribution determined by the thermal population of the excited magnetic states. We can estimate the magnetic specific heat as the difference between the total experimental heat capacity and the calculated vibrational part. Knowledge of the PDOS enables direct calculation of the lattice contribution to the specific heat by using the harmonic approximation [45]. In the harmonic approximation the lattice heat capacity at constant volume *C_V_* can be determined as follows [46]

$$C_{V}\left(T\right)=DNk_{B}\int^{\;}{\left[\frac{\frac{\hbar \omega }{2k_{B}T}}{\sin h\left(\frac{\hbar \omega }{2k_{B}T}\right)}\right]}^{2}g_{T}\left(\omega \right)d\omega ,$$

where *D* is the number of degrees of freedom in the unit cell (three in our case), and *g_T_*(*ω*) is the total PDOS. Actually, direct experimental methods typically measure the specific heat at constant ambient pressure *C_p_*. Because the specific heat is measured in a wide temperature range, the thermal expansion cannot be neglected. As was discussed in [18], from the general thermodynamic approach, *C_p_*(*T*) is always larger than *C_V_*(*T*), and for solids, the difference can be expressed as

$$C_{P}\left(T\right)-C_{V}\left(T\right)=-T\frac{\alpha^{2}V_{0}^{2}}{\frac{dV_{0}}{dP}}=\alpha^{2}BV_{0}T,$$

where α denotes the thermal expansion coefficient, *V*_0_ the molar volume, and *B* is the bulk modulus. The bulk modulus of KFeS_2_ is estimated from the second derivative of the total energy as a function of the unit cell volume by ab initio calculations: *B* = 10.1 GPa. The total energy per unit cell volume itself was obtained from a polynomial fit, which was truncated after the fourth-order term. The thermal expansion coefficient α is estimated from the crystal structure data c for room temperature and 14 K in reference [1]. Hence, *C_P_* ≈ *C_V_* + 0.003 (J/mol K^2^)*T* was used to calculate the specific heat shown in Figure 9 (see the legend).

The comparison of the calculated lattice contribution to the specific heat and experimentally obtained total specific heat is in Figure 9. Below 25 K, the calculated lattice contribution demonstrates a cubic dependence on temperature (the inset in Figure 9), which is predicted by the Debye model of the specific heat of solids.

**Figure 10:**
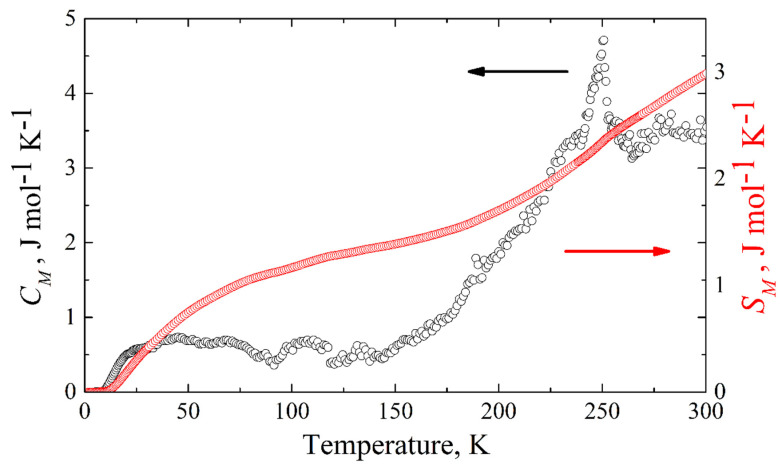
Temperature dependence of the magnetic heat capacity (black empty circles) and change of the magnetic entropy (red empty squares) of KFeS_2_ obtained as the difference between the experimentally measured specific heat and the calculated lattice contribution.

The magnetic specific heat of KFeS_2_ is shown in Figure 10. The corresponding magnetic entropy change can be calculated from the experimentally measured magnetic specific heat as $\Delta S_{M}=\int^{\;}\left[C_{m}\left(T\right)/T\right]dT$. Integrating from zero temperature to 300 K, we obtain Δ*S_M_* = 2.98 J mol^−1^ K^−1^ (see Figure 10), which is an estimation of the lower boundary, because of the temperature limit (*T* ≤ 300 K) of our equipment. For quasi-one-dimensional systems, one expects magnetic fluctuations to persist even significantly above the Néel temperature. This must be compared to the theoretical expectation for the possible cases of low-spin 1/2, intermediate spin 3/2, and high-spin 5/2 states for Fe^3+^ (3*d*^5^) atoms in KFeS_2_, where the corresponding entropy change Δ*S_M_* at the antiferromagnet-paramagnet order-disorder transition is given by *Rln*2 = 5.76 J mol^−1^ K^−1^, *Rln*4 = 11.52 J mol^−1^ K^−1^, and *Rln*6 = 14.89 J mol^−1^ K^−1^, respectively. Considering the temperature limitation of 300 K of our PPMS-9 setup, compared with the thermal stability temperature ~900–1000 K of the crystal [4], the experimentally obtained value of the magnetic entropy change Δ*S_M_* should be considered as the lower boundary, suggesting as most probable the low-spin state *S* = 1/2, but not excluding *S* = 3/2.

## 6. Conclusions

We have performed element-specific ab initio density functional theory calculations of the vibrational properties of the covalent antiferromagnetic chain compound KFeS_2_. The calculations are based on the DFT + U approach to the KFeS_2_ band structure and magnetic moment per iron ion. Experimentally, the high-quality single-crystalline KFeS_2_ samples were grown utilizing the Bridgman method. The crystal structure and absence of the alien crystallographic and magnetic phases were confirmed with the powder XRD and ^57^Fe Mössbauer spectroscopy measurements. The temperature dependence of the specific heat of KFeS_2_ was measured. Then, nuclear inelastic scattering measurements on KFeS_2_ samples were performed and partial PDOS were evaluated from the NIS spectrum. The PDOS obtained from NIS experiments was used to calibrate the frequency scale of the DFT + U calculations by comparison of the measured and calculated iron PDOS. The IR absorption measurements showed the presence of two high-frequency peaks consistent with the calculated PDOS. The ab initio results for the total PDOS with the corrected frequency scale were used to estimate the temperature dependence of the lattice specific heat of KFeS_2_. The magnetic specific heat was obtained as a difference between the experimentally measured heat capacity and the calculated lattice one in the temperature range of 2–300 K. The resulting magnetic contribution to the entropy change, Δ*S_M_*(300 K) ≈ 2.98 J mol^−1^ K^−1^, obtained by integrating the magnetic specific heat, is not far below the 5.76 J mol^−1^ K^−1^ expected for the pure low-spin state of the iron ion at the disorder-order transition. It is clear that there is enough room up to the crystal stability temperature above 900 K to collect further entropy change; however, at high temperatures, the approximations made upon the lattice specific heat calculations (harmonic vibrations, independence of the phonon eigenfrequencies on temperature, thermodynamic accounting for thermal expansion, etc.) break down. The estimation of the magnetic entropy change provides, therefore, a lower boundary for the statements about the reduced value of the iron spin state. Nevertheless, such verification of the degree of correctness of the calculations on one hand, and on the other hand, obtaining information about the possible parent spin state of the iron ion, leading to the observed magnetic moment, provides motivation for the development of advanced theoretical models of spin chains, generalizing the known Bonner-Fischer model of *S* = 1/2 linear magnetic chains with anisotropic coupling [47].

Another important result of this study is the building of a consistent picture of the physical properties of the KFeS_2_ quasi-one-dimensional compound within the DFT + U approximation. Indeed, it is well known that GGA + U functionals may underestimate bandgaps, therefore, hybrid functionals [48,49,50,51] or self-interaction-corrected DFT [52,53] techniques have been used to obtain more accurate results. We used a strategy of finding U and J (U = 1.5 eV and J = 2 eV [42]) within the DFT + U route, which provides the experimentally measured magnetic moment per iron ion (1.9 μ_B_) and band gap (0.35 eV). Then, the phonon frequencies were calculated from the eigenvalues of the dynamical matrix. The latter involves the evaluation of interatomic forces in all directions acting on atoms of supercells in the amount of 18 for the particular KFeS_2_ lattice. Thus, in our case for each supercell containing 96 atoms the dynamic matrix accumulates 96 × 18 × 3 = 5184 force constants. The resulting PDOS for iron ions is compared with the experimentally measured PDOS obtained from nuclear inelastic scattering and shows quite good agreement, thus, opening prospects for the analysis of thermal properties. The overall consistency of the calculated magnetic moment, bandgap, and iron PDOS with the experimental results make the DFT + U scheme promising for studying the physical properties of the KFeS_2_-family compound by the low-cost ab initio calculation methods in the light of recent attempts to search for high-temperature superconductivity in such compounds [54].

## Data Availability

Not applicable.
